# Frequency of hepatitis B and C in health care providers at three referral hospitals in Libya

**DOI:** 10.11604/pamj.2020.37.214.23997

**Published:** 2020-11-03

**Authors:** Abdel-Naser Elzouki, Rafat Lubbad, Islam Elzouki, Ahmed Elhaddad, Abdulfattah Ibrahim

**Affiliations:** 1 Department of Medicine, Hamad General Hospital, Hamad Medical Corporation, Doha, Qatar,; 2 Weill Cornell Medical College, Doha, Qatar,; 3 Department of Medicine, Althora Hospital, Albaida, Libya,; 4 Department of Medicine, Tripoli Central Hospital, Tripoli, Libya,; 5 Department of Medicine, Infectious Disease Unit, Benghazi Medical Center, Benghazi, Libya,; 6 Faculty of Medicine, Benghazi University, Benghazi, Libya

**Keywords:** Health care workers, hepatitis B infection, hepatitis C infection, hepatitis B vaccine, Libya

## Abstract

**Introduction:**

the aim of the present study was to determine the frequency of HBsAg and anti-HCV antibodies in health care providers (HCPs) at three referral hospitals in Libya, and to correlate the HBsAg status with history of hepatitis B vaccination among HCPs.

**Methods:**

one hundred eighty-two HCPs, with a mean age (±SD) of 32.9±8 years and age range from 20 to 59 years, were enrolled in this study. They were 50 doctors, 68 nurses, 42 laboratory technicians, 12 hospital cleaners, five anesthesia technicians and five midwives. They were tested, after obtained a written consent, for the presence of HBsAg and anti-HCV antibodies by enzyme linked immuno-sorbent assay (ELISA) techniques. A pre-test questionnaire was filled by each HCP to verify place of work, working period, type of work, status of HBV vaccination, and history of needle stick injury.

**Results:**

four HCPs have anti-HCV antibodies positive (2.2%) and nine were HBsAg positive (4.9%). Only 52% (95/182) of the HCPs received full dose of hepatitis B vaccine, while the others either not completed the vaccination schedule or have not receive it. One hundred (54.9%) of the participants had exposed to blood via needle stick injury during their work, 6 (6%) of them were HBsAg positive and three (3%) were anti-HCV positive. Needle stick injury was considered as primary risk factor in 66.7% (6/9 HCPs) of HBsAg-positives and 75% (3/4 subjects) of anti-HCV-positives.

**Conclusion:**

the present study showed a higher frequency of HBsAg than anti-HCV among HCPs in three major hospitals in Libya. This difference may be explained by the low hepatitis B vaccination rate and the high rate of needle stick injury among this high risk group for these infections.

## Introduction

Both hepatitis B and C infections are an important occupational risk for health care providers (HCPs). It is estimated that 600,000 to 800,000 needle stick injuries occur among HCPs worldwide annually, and only half of them are registered [1]. Hepatitis B virus (HBV) is the greatest threatening infection in HCPs, its risk of transmission is at least four times in HCPs than general population [2]. A recent systematic review and meta-analysis illustrated that only quarter of HCPs in Africa were fully vaccinated against HBV [3]. In contrast, a decrease in HBV infection among HCPs has been demonstrated in USA and Europe that is most probably due to the introduction of HBV vaccine [4].

The prevalence of HBsAg in western world ranges between 0.2 to 1%. At least 20-30% of HBsAg positive carriers die from complications of liver cirrhosis [5]. In Libya, the prevalence of HBsAg infection was found to be 2.2% in a large nationwide general population-based seroprevalence survey of more than 65000 randomly selected participants [6], and it remains the leading cause of liver cirrhosis including hepatocellular carcinoma [7]. Hepatitis C has become a curable disease with the use of direct antiviral agents (DAAs) [8,9]. Worldwide, more than 170 million individuals have hepatitis C virus (HCV) infection [10], of whom 71 million have chronic infection [11]. A recent systematic review demonstrated that most countries in North African region have low to moderate prevalence of anti-HCV antibodies (0.1% to 6.6%) [12], with exception of Egypt in which the overall prevalence of HCV is 16.8% [12]. Libya has a reported HCV prevalence of 1.3% among general population [6]. Assessment of transmission risk of blood borne pathogens to HCPs requires information derived from various sources, factors influencing the risk of transmission to individual HCP includes place of work, working period, type of occupation, and rate of exposure to blood, blood products and serous fluids of the patients [13]. It is also influenced by the prevalence of HBV and HCV infection in the served population. Furthermore, transmission of HBV and HCV in HCPs occurs predominantly through percutanuos or mucosal exposure of the HCPs to blood or body fluids of the infected patients. Needle stick injury is the most common route for transmission of these infections from infected blood to the HCPs.

Prospective studies have estimated the average risk of HBV transmission to HCPs is 6% to 30%, while in HCV is 1.8% after percutaneous exposure [14]. There is growing evidence of literature on HBV and HCV in Libya, however, there limited information on prevalence and associated risk factors among HCPs [15]. The aim of the present study was to determine the magnitude of the transmission risk of hepatitis B and hepatitis C to HCPs in three major referral hospitals in eastern Libya, and to evaluate how the risk of transmission is influenced by working period, place of work, occupation, vaccination against HBV and prevalence of needle stick injury in HCPs.

## Methods

**Setting and study population**: the present study was conducted in three major referral hospitals in eastern Libya: 1) Aljomhoria hospital, a 515 bed teaching hospital in Benghazi city; 2) 7^th^ October hospital, a 250 bed teaching hospital in Benghazi city, and Althoura hospital, a 480 beds teaching hospital in Albaida city. A total of 182 HCPs were randomly enrolled from different places of work and with different hospital job duties as doctors, nurses, help nurses and laboratory technicians.

**Data collection**: a pre-test self-administered structured questioner was used to collect data from each HCP on demographic characteristics, type of work and place of work in the hospital, working period, history of needle stick injury, history and duration of hepatitis B vaccination. Data collection from HCPs was facilitated by two staff members (one HCP who was not included in the study and a laboratory professional for blood collection) at each hospital.

**Serological analysis**: after consent was taken from each subject, blood samples were collected and transported to the Central Laboratory in Aljomhoria hospital using a cold chain. The blood samples were centrifuged and stored in -20°C until tested by enzyme linked immunosorbent assay (ELISA) techniques for detection of hepatitis B surface antigen (HBsAg) and anti-hepatitis C (anti-HCV) antibodies. Samples found positive at initial screening were analyzed again for the second time. All the samples found positive initially were found positive on repeated analysis.

**Data analysis**: descriptive statistics were used to describe the data. For continuous variables, the mean and standard deviation (SD) were used to summarize the data. For categorical variables, frequencies and percentages were reported. The differences between groups were analyzed using Pearson's chi-squared test or Fisher's exact test as appropriate. A p-value of <0.05 was considered statistically significant. The data were processed and analyzed using SPSS Statistics (SPSS Statistics Inc., Chicago, US, version 20).

**Ethical considerations**: the ethical clearance was secured from Aljomhoria Teaching Hospital, Benghazi University. The objective of the study was explained to each study participant. The participants were informed that there are interviews and blood sample collection for HBV and HCV screening. Written informed consent for participation was obtained from each participant. The participation in the study was on a voluntary basis and the participants could withdraw from the study during the interview. Participants were ensured that all collected data will be used only for the research purpose. Test results were kept confidential by using a unique code given to each participant. Laboratory staff has access only to the unique codes written on the test tubes. Only two of the research team members (RL and AE) had access to both the unique codes and participants' identifications written in a separate format. All participants were informed of the result and those with a positive result were counseled and linked to care.

## Results

A total of 182 HCPs from three major referral hospitals in eastern Libya were enrolled in the present study. The distribution of HCPs according to the three hospitals was 83 HCPs from Aljomhoria hospital, Benghazi; 57 HCPs from 7^th^ October hospital, Benghazi; and 42 HCPs from Althoura hospital, Albaida. There were 143 (78.6%) females and 39 (21.4%) males. The mean age was 32.9 ± 8.0 years with range from 20 to 59 years. Table 1 shows age distribution of the studied HCPs, 84% of them were below the age of 40 years. Four (2.2%) HCPs were anti-HCV antibodies positive (three females and one male), and 9 (4.9%) HCPs were HBsAg positive (6 females and 3 males). The frequency of HBsAg positivity was higher among the HCPs at Aljomhoria Hospital (6%) than Althoura Hospital (4.8%) and 7^th^ October Hospital (3.5%) (Table 2). One hundred (55%) of the HCPs were exposed to blood through at least one needle stick injury during their work, 6 (6%) of them were HBsAg positive and three (3%) were anti-HCV positive. Needle stick injury was considered as a primary risk factor in 66.7% (6/9 subjects) of HBsAg positives and 75% (3/4 subjects) of anti-HCV positives.

**Table 1:** age distribution of the studied health care providers in the three hospitals of eastern Libya (N=182)

Percentage (%)	Numbers	Age groups (years)
39.6	72	20-29
43.4	79	30-39
11.5	21	40-49
5.5	10	50 -59

**Table 2:** distribution of HBsAg positivity of health care professionals according to hospital location, type of work and hepatitis B vaccination status

Characteristic	HbsAg+ ve (N=9)		HbsAg-ve (N=173)	
	Number	Percentage (%)	Number	Percentage (%)
**Hospital name**				
Aljomhoria Hospital (N= 83)	5	6.0	78	94.0
7^th^ October Hospital (N= 57)	2	3.5	55	96.5
Althoura Hospital (N= 42)	2	4.8	40	95.2
**Occupation**:				
Doctors (N=50)	2	4.0	48	96.0
Laboratory Technicians (N=42)	3	7.1	39	92.9
Nurses (N=68)	3	4.4	65	95.6
Cleaners (N=12)	1	8.3	11	91.7
Anesthesia Technicians (N=5)	0	0	5	100
Midwives (N=5)	0	0	5	100
**Place of work:**				
Laboratory (N=42)	3	7.1	39	92.2
Labour Room (N=33)	1	3.0	32	97.0
Dialysis Unit (N=16)	1	6.2	15	93.8
Operation Room (N=42)	1	2.4	41	97.6
Endoscopy Unit (N=4)	1	25.0	3	75.0
Emergency Department (N=19)	1	5.3	18	94.7
In-patient words (N=16)	1	6.2	15	93.8
Hospital Administrators (N=10)	0	0	10	100
**Vaccine doses received:**				
One dose (N= 13)	1	7.7	12	92.3
Two doses (N= 24)	1	4.2	23	95.8
Three doses (N= 95) ^*^	0	0	95	100
Vaccination not received (N= 50) ^**^	7	14.0	43	86.0

* p= 0.01, ^**^p= 0.02

Of the nine HBsAg positive HCPs, 7 subjects (77.8%) had worked for a period of more than 5 years compared to two subjects (22.2%) had worked for a period of less than 5 years (P=0.03). Of the 4 anti-HCV positive HCPs, three subjects (75%) had worked more than 5 years while the other one (25%) from this group worked less than 5 years. The distribution of HBsAg positivity according to the type of work (occupation) of the studied HCPs is; two doctors (4%), three lab technicians (7.1%), three nurses (4.4%) and one hospital cleaner (8.3%) (Table 2). The four anti-HCV positive HCPs distributed as one doctor and three nurses. The distribution of the HBsAg positivity according to the place of work of the HCPs is shown in Table 2. Two of the anti-HCV positive HCPs were working in the operation theater, one in the in-patient wards and one in the Emergency Department. Only 52.2% (95/182) of the HCPs received full dose of HBV vaccine, while the remaining 47.8% (87/182) were either not completed the vaccination schedule (37 subjects) or not received it at all (50 subjects) (Table 2). None of the 95 HCPs who had received complete HBV vaccination were HBsAg positive.

## Discussion

HCPs worldwide face the risk of occupational infections by blood borne pathogens, including HBV, HCV and human immunodeficiency virus (HIV). Infection with these viruses is an important occupational hazard for HCPs. In the present study, attempts have been made to assess the status of infection associated with occupational exposure to HBV and HCV in three tertiary health care settings in eastern Libya. Although vaccination against HBV was introduced in the health care system in Libya since early nineties, significantly high frequency of HBsAg (4.9%) was found among HCPs compared to what was reported in general population (2.2%) in a large population-based study from Libya [6]. In contrast, the frequency of Anti-hepatitis C antibodies (2.2%) was slightly higher to the frequency observed among the general population of Libya (1.3%) [6]. The frequency of HBsAg was high comparing to the previous studies reported from neighboring countries (i.e., Tunisia 2% and Egypt 1.4%) and developed countries (0.6% to 2.2%) [16-18].

The reasons for this substantial variation in transmission rates have not been determined; probably infection control might be suboptimal in these hospitals and primary prevention strategies including prompt reporting and management of occupational exposures were lacking. Average blood volume inoculated during needle stick injury with 22 gauge needle is approximately one microlitre; a quantity sufficient to contain up to 100 infective doses of HBV [19,20]. In case series study of HBV infected HCPs less than 10% recalled history of needle stick injury or percutaneous exposure to blood and 29-38% recalled caring of HBsAg positive patients in the last six months prior to test [21]. Post-exposure prophylaxis with hepatitis B Immunoglobulin (HBIG) and/or HBV vaccine should be used when indicated (e.g., after percutaneous or mucous membrane exposure to blood known or suspected to be HBsAg positive). Needle stick or other percutaneous exposures of unvaccinated HCPs should lead to initiation of the HBV vaccine series regardless of the HBV status of the source patient [22]. Post-exposure prophylaxis should be considered for any percutaneous, ocular, or mucous membrane exposure to blood in the workplace and is determined by the HBsAg status of the source and the vaccination and vaccine-response status of the exposed person [22]. The results of the present study highlight the need of widespread adoption of needle stick prevention program in health care settings to reduce the risk of the occupational infection for HCPs in these three major hospitals and probably in the other hospitals in Libya.

In the present study, the frequency of positive-HBsAg was more than the frequency of positive-anti-HCV antibody in enrolled HCPs. This difference is possibly due to the HCPs were not taken or completed their compulsory HBV vaccination (Only 52% did), and/or carelessness of the HCPs about accidental needle sticking or might be some places at which the study was done have higher prevalence of HBsAg positive population than others. In general, the prevention of occupational infection by blood-borne viruses relies on avoiding exposure and receiving immunization against HBV and post-exposure prophylaxis. Efforts to reduce the risk of occupational exposure by strictly implementing standard precautions while handling all hospital attendances are essential to minimize transmission rate [23]. These precautions consist of wearing gloves, gowns and eye protection or a face shield that should be worn during procedures and patient care activities that are likely to have splashes and sprays of blood, body fluids, secretions, and excretions to the HCPs as well as implementation of safe injection practices. The findings of this study highlight the need for strict infection control measures in participant´s health care facilities.

## Conclusion

It is clear that HCPs are at higher risk for HBV and HCV infection and the frequency of HBsAg is higher than anti-HCV in those working at high risk places such as laboratories and hemodialysis units. Therefore, the recommended standard precautions should be used during caring any patient and HBV vaccination must be given to all HCPs at risk. There should be counseling of HCPs about the accidental exposure if happened and post exposure prophylaxis which will help to prevent transmission of blood borne infections in workplace.

### What is known about this topic

- In Libya, the prevalence of HBV and HCV infection was found to be 2.2% and 1.3%, respectively, in a large nationwide population-based seroprevalence survey of more than 65000 randomly selected participants;
- Health care providers are at high risk of hepatitis B and C infection due to occupational exposure to blood and body fluids;
- Vaccination against HBV is an effective tool of preventing the transmission of HBV among high risk groups including health care providers.

### What this study adds

- The present study demonstrated a higher frequency of HBsAg (4.9%) than anti-HCV (2.2%) among HCPs in three tertiary hospitals in eastern Libya. This difference may be explained by the low hepatitis B vaccination rate and the high rate of needle stick injury;
- In Libya, there is a need of widespread adoption of needle stick prevention program in health care settings to reduce the risk of hepatitis B and C infection for HCPs;
- The recommended standard precautions should be used during caring any patient and compulsory HBV vaccination must be given to all HCPs at risk in Libya.
